# Trends in Out-of-Pocket Costs for Naloxone by Drug Brand and Payer in the US, 2010-2018

**DOI:** 10.1001/jamahealthforum.2022.2663

**Published:** 2022-08-19

**Authors:** Evan D. Peet, David Powell, Rosalie Liccardo Pacula

**Affiliations:** 1 RAND Corporation, Pittsburgh, Pennsylvania; 2 RAND Corporation, Arlington, Virginia; 3 University of Southern California, Los Angeles

## Abstract

**Importance:**

Improving access to naloxone is a critical component of the nation’s strategy to curb fatal overdoses in the opioid crisis. Standing or protocol orders, prescriptive authority laws, and immunity provisions have been passed by states to expand access, but less attention has been given to potential financial barriers to naloxone access.

**Objective:**

To assess trends in out-of-pocket (OOP) costs for naloxone and examine variation in OOP costs by drug brand and payer.

**Design, Setting, and Participants:**

This observational study analyzed US naloxone claims data from Symphony Health and associated OOP costs for individuals filling naloxone prescriptions by drug brand and payer between January 1, 2010, to December 31, 2018. The data were analyzed from March 31, 2021, to April 12, 2022.

**Main Outcomes and Measures:**

The main measures were trends in annual number of naloxone claims (overall, by payer, and by drug brand) and mean annual OOP costs per claim (overall, by payer, and by drug brand).

**Results:**

Of 719 612 naloxone claims (172 894 generic naloxone, 501 568 Narcan, and 45 150 Evzio) for 2010 through 2018, the number of naloxone claims among insured patients began rapidly increasing after 2014; at the same time, the mean OOP cost of naloxone increased dramatically among the uninsured population. Comparing 2014 with 2018, the mean OOP cost of naloxone decreased by 26% among those with insurance but increased by 506% among uninsured patients. For the uninsured population, the impediment of cost was even larger for certain brands of the drug. In 2016, the mean OOP cost for Evzio among uninsured patients rose to $2136.37 (a 2429% increase relative to 2015) compared with the mean cost of generic naloxone, $72.88, and the cost of Narcan in its first year, $87.95. Throughout the period, the mean OOP costs paid by uninsured patients were higher for Evzio at $1089.17 (95% CI, $884.17-$1294.17) compared with $73.62 (95% CI, $69.24-$78.00) for Narcan and $67.99 (95% CI, $61.42-$74.56) for generic naloxone.

**Conclusions and Relevance:**

In this observational study, the findings indicated that the OOP cost of naloxone had been an increasingly substantial barrier to naloxone access for uninsured patients, potentially limiting use among this population, which constituted approximately 20% of adults with opioid use disorder.

## Key Points

**Question:**

How have out-of-pocket (OOP) costs for naloxone in the US changed between 2010 and 2018 overall and by drug brand and payer?

**Findings:**

This observational study of 719 612 pharmacy claims data shows that OOP costs of naloxone grew substantially beginning in 2016. However, OOP costs did not increase for all patients and all brands of naloxone but primarily for uninsured patients and for the Evzio brand.

**Meaning:**

The findings suggest that the OOP cost of naloxone has been an increasingly substantial barrier to naloxone access for uninsured patients, a population that constitutes nearly one-fifth of adults with opioid use disorder.

## Teaser

This observational study assesses trends in out-of-pocket costs for naloxone in the US between 2010 and 2018 and examines variation by drug brand and payer.

## Introduction

Throughout the past 2 decades, opioid-involved drug overdoses have continued to rise,^1^ with overdose deaths hitting a historic high in 2020^2^ and continuing to grow throughout the COVID-19 pandemic.^3^ A critical component of the nation’s strategy to reverse these persistent trends is naloxone. Expanding the distribution of naloxone, a prescription opioid antagonist medication that can counter the respiratory effects of an opioid overdose and prevent death, has been a primary objective of the efforts to combat the opioid crisis of multiple presidential administrations.^4,5^ To expand access, states have passed a flurry of laws, such as standing or protocol orders,^6^ prescriptive authority laws,^7^ and immunity provisions for health care professionals and laypeople.^8^ Existing evidence indicates that these policies have substantially increased naloxone dispensing through pharmacies,^9,10,11^ and this greater access has reduced mortality.^7,12,13,14^ However, despite the growing number of naloxone access laws, recent studies show that naloxone distribution remains insufficient.^15^ Despite guidelines promoting opioid/naloxone coprescribing, naloxone prescribing rates are low (below 3%) even for those receiving long-term opioid therapy.^16^

Although there has been considerable attention on the legal barriers to naloxone distribution, far less attention has been given to the financial barriers of expanding access, particularly to individuals (carriers) likely to be in a position to help someone who has overdosed. The out-of-pocket (OOP) cost of naloxone is an important determinant of access, and there is evidence that naloxone demand responds to price.^17^ Like any pharmaceutical product, naloxone OOP costs vary by insurance status. Approximately 20% of those with opioid use disorder are uninsured,^18^ making reported price increases a major cause of concern among academics^19^ and policy makers.^20^

The objective of this study was to assess trends in naloxone OOP costs between 2010 and 2018. We do so by documenting nationwide trends and by examining variation in OOP cost trends by drug brand and payer.

## Methods

### Data Sources

In this observational study, we used nationwide prescription claims data for the period 2010 to 2018 from Symphony Health. We followed the Strengthening the Reporting of Observational Studies in Epidemiology (STROBE) reporting guideline. This study was approved by RAND’s Human Subjects Protection Committee (which is equivalent to an institutional review board).

 Symphony Health is a health technology company that compiles prescription claims data from retail pharmacies. These data describe a 72% sample of retail pharmacies in the US and approximately 90% of prescriptions filled in those pharmacies.^21^ The data originate from payers and processors of prescription drug claims and describe prescriptions to all payer types—private insurance, Medicare, Medicaid (fee-for-service and managed care), other public assistance programs (US Department of Veterans Affairs [VA]/Tricare), as well as uninsured patients. The data describe the number of claims and OOP costs aggregated to the 3-digit zip code, year-quarter, drug brand–dosage, and payer type. Drug brand, which consists of generic naloxone (0.4-mg and 1-mg doses), Evzio (0.4-mg and 2-mg doses), and Narcan (4-mg doses), directly maps to route of administration. Generic naloxone during the 2010 to 2018 period could only be administered through injection. Evzio is an autoinjector and operates similar to an epinephrine injection with different specified doses. Narcan is administered intranasally and was the first formulation that did not require injection.

### Sample Description

We used 2010 to 2018 naloxone prescription claims data with claims counts by insurance type and brand. We aggregate these claims data to the national, annual level. To calculate claims rates for each payer type’s beneficiary population, we used national, annual beneficiary counts by payer type (private, Medicare, Medicaid, other public assistance, and uninsured) from the Kaiser Family Foundation data as the denominator.^22^

### Key Variables

We created annual counts of naloxone claims within a calendar year overall. We then calculated annual rates of naloxone claims by payer by dividing the payer-specific counts by the payer-type beneficiary population and then scaling by 100 000. Similarly, we created annual totals of OOP costs paid during a calendar year, expressed in nominal dollars. We then calculated annual mean OOP costs per claim by dividing by the total number of naloxone claims in the same year. These same calculations were performed by payer and by drug brand.

### Statistical Analysis

We examined national-level trends in naloxone claims and OOP costs during the study time frame, 2010 to 2018. The graphs presented in this study convey the annual mean OOP costs overall, by payer, and by drug brand, and the annual number of naloxone claims overall, by payer, and by drug brand. All costs are measured in nominal dollars. Confidence intervals for OOP costs were calculated from the level of 3-digit zip code, year-quarter, drug brand–dosage, and payer type data. We present the 2.5 and 97.5 percentiles of this distribution as the CIs.

The data were analyzed using Stata statistical software, version 16.1 (StataCorp LLC). This descriptive study did not involve statistical tests. This study was conducted from March 31, 2021, to April 12, 2022.

## Results

A total of 719 612 naloxone claims (172 894 generic naloxone, 501 568 Narcan, and 45 150 Evzio) were assessed between 2010 and 2018. In 2010, 11 432 naloxone claims were observed in the data, all representing generic naloxone. A total of 5613 were paid by private insurers, 2216 were paid by Medicare, 8 by Medicaid, none by VA/Tricare, and 3595 were paid fully OOP by uninsured patients. In contrast, by 2018 there were 386 249 claims in the data for all types of naloxone, of which 29 395 (7.6%) were generic naloxone claims, 8632 (2.2%) were Evzio claims, and 348 222 (90.2%) were Narcan claims. In that same year, 119 886 claims were paid by private insurers, 132 370 claims were paid by Medicare, 118 881 were paid by Medicaid, 6303 were paid by VA/Tricare, and 8809 were paid fully OOP by the uninsured population.

**Figure 1.**
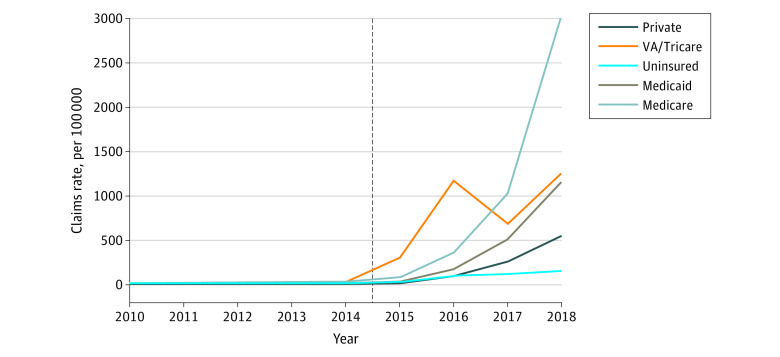
Naloxone Claims per 100 000 by Payer Between 2010 and 2018 Claims rate is defined as the number of naloxone claims in each year per beneficiary population scaled by 100 000. VA indicates Veterans Affairs.

The rate of naloxone claims began a rapid expansion in 2015 and 2016, coinciding with the passage of many state naloxone access laws^7^ and the approval of Narcan in November 2015^23^ (Figure 1). However, payer type played an important role in the increased access to naloxone. At first, the rate of naloxone claims among the VA/Tricare population grew most rapidly, reaching 1184 per 100 000 beneficiaries in 2016, before dropping by a third in 2017 and then recovering to 1261 claims per 100 000 beneficiaries in 2018. Naloxone claims grew fastest among Medicare beneficiaries, exceeding 3000 claims per 100 000 beneficiaries annually by 2018. While coprescribing was not observed in the data, this growth coincided with a rise in coprescribing among Medicare enrollees found in other work.^24^ The rate of naloxone claims among Medicaid beneficiaries grew more slowly but by 2018 was comparable with that of VA/Tricare, specifically 1166 per 100 000 beneficiaries. The rate of naloxone claims among private insurance beneficiaries increased even slower but by 2018 was 548 per 100 000 beneficiaries. The slowest growth in the rate of naloxone claims occurred among uninsured patients: by 2018, there were 154 naloxone claims per 100 000 persons without insurance. In 2014, the rate of naloxone claims dispensed through pharmacies among the insured population was 2.0 times that of the uninsured population. By 2018, the rate of naloxone claims dispensed through pharmacies among the insured population was 5.2 times that of the uninsured population.

As suggested by the Table, there may be an association between the slow growth in the rate of naloxone claims among the uninsured population and cost. In 2014, the mean OOP cost per naloxone claim was $27.97 (95% CI, $23.96-$31.98) for private insurance beneficiaries; $8.11 (95% CI, $6.03-$10.19) for Medicare beneficiaries; $3.14 (95% CI, $1.97-$4.30) for Medicaid beneficiaries; $103.07 (95% CI, $88.35-$117.78) for VA/Tricare beneficiaries; and $35.39 (95% CI, $17.60-$53.17) for uninsured patients. Among the entire insured population, the mean OOP cost per naloxone claim was $28.46 (95% CI, $24.26-$32.66). So, naloxone was more expensive for uninsured patients by $6.93 on average. Then, between 2015 and 2018, OOP costs changed dramatically. While the mean OOP cost among private insurance and Medicare beneficiaries increased marginally, the mean OOP cost for Medicaid and VA/Tricare beneficiaries dropped. The decrease for the VA was substantial: 29.49%. In total, the mean OOP cost per naloxone claim for the insured declined by 26.15%. Simultaneously, among the uninsured population, the mean OOP cost per naloxone claim increased by 506.33%.

**Table.** Difference in the Mean Out-of-Pocket Cost of Naloxone in 2014 and 2018 by Payer

Payer/year	Claims, No.	Mean (95% CI), $
Private		
2014	5637	27.97 (23.96-31.98)
2018	119 886	35.13 (34.29-35.96)
Medicare		
2014	2280	8.11 (6.03-10.19)
2018	132 370	14.11 (13.82-14.41)
Medicaid		
2014	783	3.14 (1.97-4.30)
2018	118 881	2.85 (2.70-3.00)
VA/Tricare		
2014	750	103.07 (88.35-117.78)
2018	6303	72.67 (67.35-77.98)
Uninsured		
2014	7183	35.39 (17.60-53.17)
2018	8809	249.97 (215.32-284.61)

Abbreviation: VA, Veterans Affairs.

**Figure 2.**
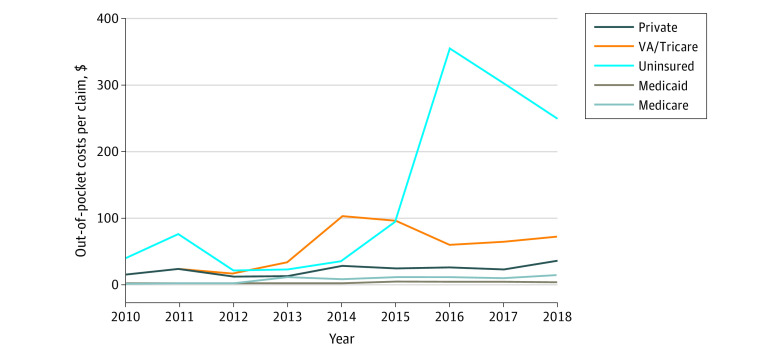
Trends in Naloxone Out-of-Pocket Costs by Payer Mean out-of-pocket costs in nominal dollars are defined by the total number of out-of-pocket costs per year divided by the beneficiary population. VA indicates Veterans Affairs.

Figure 2 describes the annual mean OOP cost per claim by payer for the 2010 to 2018 period (see also eTable 1 in the Supplement). The uninsured population paid higher mean OOP costs than the beneficiaries of any other payer in every year except 2013 and 2014, when VA/Tricare beneficiaries paid higher OOP costs. However, the differences in OOP costs between the insured and uninsured grew substantially starting in 2015. The mean OOP cost per naloxone claim rose to a peak of $355.08 among the uninsured population in 2016 before declining to $249.97 in 2018. The mean OOP costs paid by VA/Tricare also varied year by year, ranging from $17.49 (2012) to $103.07 (2014). The mean OOP costs paid by the beneficiaries of other payers were lower and varied much less: private, from $11.98 (2012) to $35.12 (2018); Medicare, from $0.78 (2010) to $14.11 (2018); and Medicaid, from $0.15 (2010) to $8.75 (2012).

**Figure 3.**
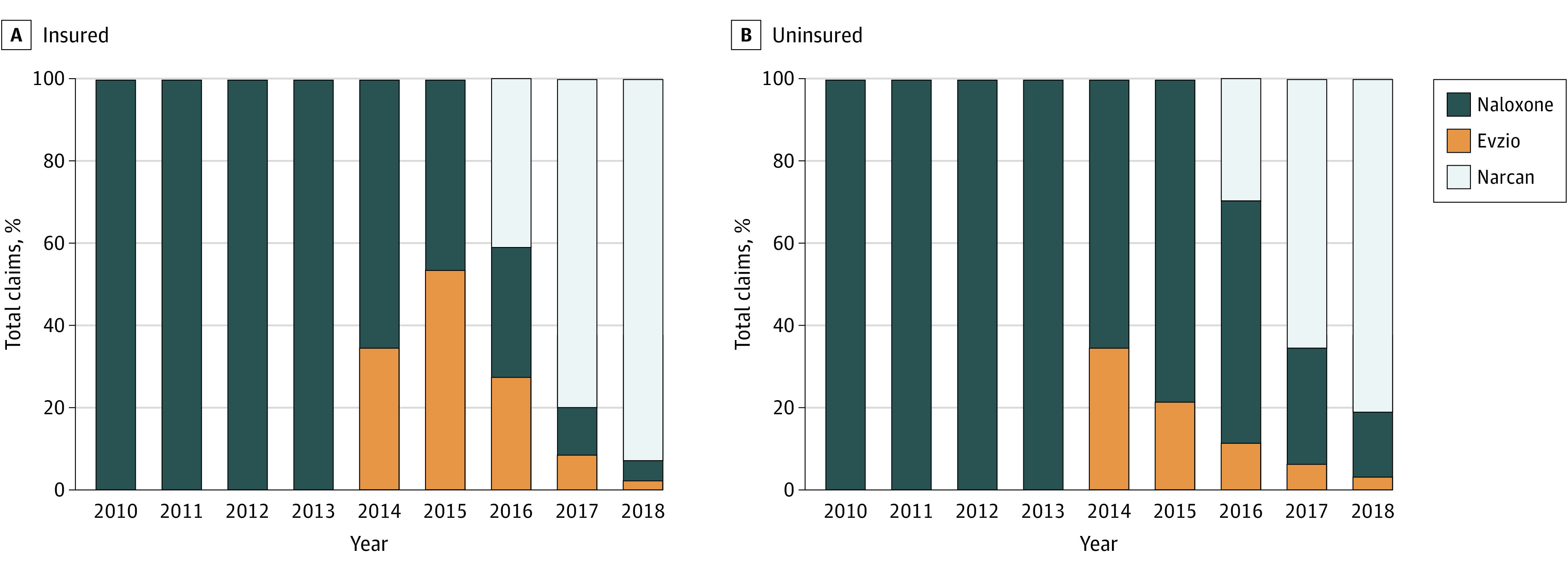
Share of Naloxone Claims by Drug Brand Among Insured and Uninsured Populations, 2010-2018 The graphs include the share percentages for Evzio, generic naloxone, and Narcan, corresponding to each year in which the drugs were available among the insured population (A) and uninsured population (B).

The market share of each drug brand changed dramatically throughout the 2010 to 2018 period. Figure 3 shows the annual share of naloxone claims by drug brand for insured and uninsured patients. Early in the period, only generic naloxone was available, and very few claims were made. Evzio entered the market in 2014 and immediately captured 34% of the total market. Evzio’s share of the insured market grew to 53% in 2015, while its share of the uninsured market grew to 21%. In 2016, Evzio’s market share began to decline after Narcan was introduced. Narcan immediately captured 41% of the insured market and 30% of the uninsured market, while Evzio declined to 27% of the insured market and 11% of the uninsured market. Generic naloxone’s share of the insured market in 2016 dropped to 32% but remained dominant in the uninsured market, 59%. However, by 2017, the majority of all naloxone claims were for Narcan: 80% of insured, 65% of uninsured. By 2018, 92% of insured and 81% of uninsured claims were for Narcan, with the second most common of both insured and uninsured claims being generic naloxone, followed by Evzio.

**Figure 4.**
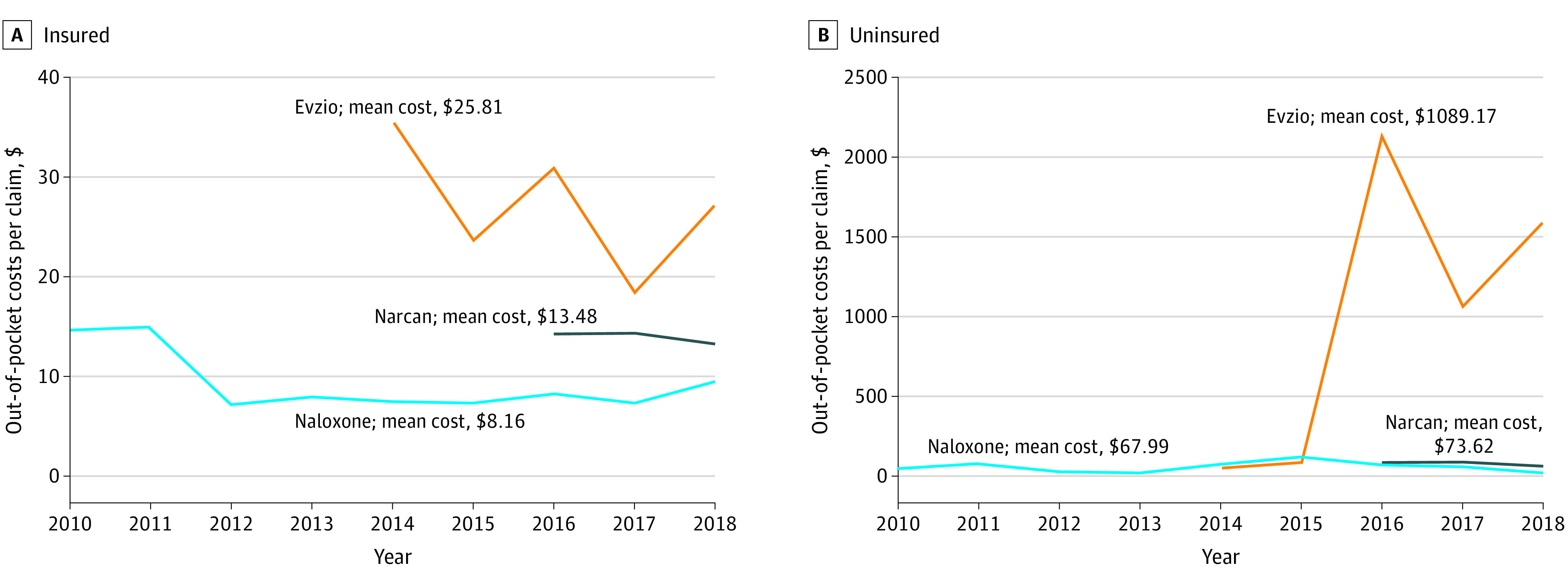
Trends in Out-of-Pocket Costs by Drug Brand Among Insured and Uninsured Populations, 2010-2018 Mean out-of-pocket costs in nominal dollars are defined by the total number of out-of-pocket costs per year divided by the beneficiary population among the insured population (A) and the uninsured population (B).

Although brand discrepancies in mean OOP costs were present throughout the period for both insured and uninsured claims, the cost discrepancies among insured patients were dwarfed by the disparate costs paid for each drug brand by uninsured patients (Figure 4; eTables 2 and 3 in the Supplement). Among insured patients, the mean OOP cost of Evzio (2014-2018) was $25.81, 1.91 times higher than Narcan ($13.48) and 3.16 times higher than generic naloxone ($8.16). However, among the uninsured population, the mean OOP cost per claim of Evzio was $1089.17 (95% CI, $884.17-$1294.17), compared with $73.62 (95% CI, $69.24-$78.00) per Narcan claim and $67.99 (95% CI, $61.42-$74.56) per generic naloxone claim. In other words, for uninsured patients, the mean OOP cost of Evzio was 14.79 times the mean OOP cost of Narcan and 16.02 times the mean OOP cost of generic naloxone. However, Evzio was not always dramatically more expensive than the other options for uninsured patients. In 2014 and 2015, the mean OOP cost for Evzio among the uninsured population was $51.75 and $84.74, respectively. In contrast, in the same years, the mean OOP cost for generic naloxone among the uninsured population was $73.89 (2014) and $122.23 (2015). In 2016, the mean OOP cost for Evzio among the uninsured population rose to $2136.37 (a 2429% increase relative to 2015) compared with the mean cost of generic naloxone, $72.88, and the cost of Narcan in its first year, $87.95.

## Discussion

The results of this observational study provided evidence that though naloxone access has improved, OOP costs remain a notable impediment, particularly for uninsured patients. Naloxone claims grew substantially following the passage of naloxone distribution laws in many states and the introduction of Narcan. Narcan’s nasal spray technology made naloxone substantially easier to administer and broadened its accessibility. However, despite these legal and pharmaceutical innovations, the increase in claims was not equally distributed. Although naloxone distribution rose substantially among those with Medicare VA/Tricare and Medicaid insurance, and even among those with private insurance starting in 2017, naloxone access among the uninsured population has not experienced proportional improvements.

The Centers for Disease Control and Prevention estimates that there is only 1 naloxone prescription per 69 high-dose opioid prescriptions.^25^ Prior to this study, there was limited evidence on the financial barriers of expanding access to naloxone,^26^ and much of the policy response has focused on reducing legal, not monetary, barriers. This study provides evidence that the price of naloxone is almost certainly an impediment to more widespread adoption among uninsured patients, a vulnerable and critical population representing approximately 20% of adults with opioid use disorder^18^ and 30% of opioid overdose deaths.^27^ In 2014, the rate of naloxone claims dispensed through pharmacies among the insured population was 2 times that of the uninsured population. By 2018, the rate of naloxone claims dispensed through pharmacies among the insured population was 5.2 times that of the uninsured population. This growing disparity in claims rate was accompanied by a divergence in OOP costs. In 2014, naloxone was $6.93 more expensive for uninsured patients: $35.39 compared with $28.46 for insured patients. However, between 2015 and 2018, the mean OOP cost per naloxone claim for the insured population declined by 26.15% while increasing 506.33% for the uninsured population. Overall, OOP costs have increased for naloxone claims, which can be explained by claims purchased by uninsured patients. During this same time period, total OOP prescription drug costs in the US across all drugs declined despite an increase in total spending on pharmaceutical products.^28^

Additionally, because patients may have limited or no control over the brand of drugs they are offered by the pharmacy,^29^ the brand of naloxone they are offered may play an important role in their uptake. For uninsured patients between 2015 and 2018, the mean OOP cost of Evzio was 14.79 times more than the mean OOP cost of Narcan and 16.02 times more than the mean OOP cost of generic naloxone, though Evzio’s market share declined substantially during this time and Evzio and its generic equivalents were discontinued in 2020 after this study’s sample period ended. Although some patients have paid these high prices, it is unclear how many patients without insurance were deterred from purchasing a lifesaving dose of naloxone.

Policy makers seeking to further expand access to naloxone, particularly among the uninsured and vulnerable populations, need to pay greater attention to the OOP cost of naloxone, at least for socioeconomically disadvantaged groups.^30^ Evidence suggests that substantial improvements can be made to increase the low rates of coprescribing of naloxone to patients with opioid use disorder diagnosis or other factors associated with an increased likelihood of overdose.^31^ However, the effects of any recommendations and/or laws will be muted if patients cannot afford to pay for the drugs. Potential avenues for addressing the high OOP cost among the uninsured population include requiring pharmacies to maintain a stock of generic naloxone so it is available to uninsured patients, providing direct funding to cover the OOP cost of naloxone distributed by pharmacies to the uninsured population, adopting co-pay support to uninsured patients following the model of the Ryan White HIV/AIDS Program, and regulating prices more directly.^32^

### Limitations

The limitations of this study are as follows. First, the Symphony Health data were missing prescriptions filled in approximately 28% of retail pharmacies in the US. However, we have compared the Symphony Health data with IQVIA data representing 90% of prescriptions filled at retail pharmacies. The IQVIA data did not contain OOP cost information, but comparing the 2 data sources revealed nearly identical trends and a correlation of approximately 0.90 in the annual number of claims. Given this correspondence across data sources, we believe it is unlikely that the pharmacies missing from the sample would have dramatically influenced mean OOP costs. Second, most naloxone is not dispensed through pharmacies but, instead, to hospitals, community-based programs, first responders, and other organizations. Although the exact share is difficult to calculate, the US Food and Drug Administration estimates that approximately 83% of naloxone was sold to nonretail settings in 2017.^33^ The small share of naloxone dispensed through pharmacies, however, was a major motivation of this study because price may be the critical barrier preventing higher rates of pharmacy distribution. Unfortunately, we did not observe naloxone dispensing from nonpharmacy settings. It is possible that naloxone dispensed in nonretail settings disproportionately targets the uninsured population, so these results should be interpreted only in the context of pharmacy distribution. Third, we did not have longitudinal individual-level data, so we could not observe repeat purchases by the same individual. Also, we did not observe individual-level changes in insurance coverage. Changes in the insurance composition of the population, such as Medicaid expansions, may have partially explained changes in the relative share of fills for each insurance type. Finally, we only observed dispensing. We did not observe use or measures of intended use.

## Conclusions

In this observational study, we found a large increase in access to naloxone through pharmacies beginning in 2015. Federal and state policies have regularly targeted legal barriers to access with less emphasis on financial barriers. In April 2019, the US Food and Drug Administration approved the first generic naloxone nasal spray with the hope that its introduction would reduce cost barriers to naloxone access.^34^ Whether this additional competition drives down OOP costs is currently unknown. Future developments in naloxone access policies should consider protections for uninsured patients, who, as this study shows, have faced substantially higher OOP costs. Additionally, policy makers could consider implementing broad price subsidies for naloxone purchases, regulating co-pays for insured patients, and issuing coupons targeting uninsured patients. Reducing OOP costs could potentially increase naloxone purchasing.^17^

## Notes

Supplement. **eTable 1.** Trends in naloxone out-of-pocket costs by payer **eTable 2.** Trends in out-of-pocket costs by drug brand between 2010 and 2018 among the insured population **eTable 3.** Trends in out-of-pocket costs by drug brand between 2010 and 2018 among the uninsured population
