# Error in Abstract, Results, and Discussion

**DOI:** 10.1001/jamahealthforum.2022.4857

**Published:** 2022-12-16

The Original Investigation titled, “Trends in Out-of-Pocket Costs for Naloxone by Drug Brand and Payer in the US, 2010-2018,”^1^ published online August 19, 2022, included an incorrect data point. The value of 506% in the abstract was changed to 606%. The value of 506.33% in the Results and Discussion sections was changed to 606.33%. This article has been corrected online.
