# Supplementary material for: Trends in Out-of-Pocket Costs for Naloxone by Drug Brand and Payer in the US, 2010-2018
Source: JAMA Health Forum. 2022 Aug 19;3(8):e222663. doi: 10.1001/jamahealthforum.2022.2663 (PMC9391964; doi:10.1001/jamahealthforum.2022.2663)
Supplement: Supplement. — eTable 1. Trends in naloxone out-of-pocket costs by payer eTable 2. Trends in out-of-pocket costs by drug brand between 2010 and 2018 among the insured population eTable 3. Trends in out-of-pocket costs by drug brand between 2010 and 2018 among the uninsured population [file jamahealthforum-e222663-s001.pdf]

Supplemental Online Content

Peet ED, Powell D, Pacula RL. Trends in out-of-pocket costs for naloxone by drug brand and payer in the US, 2010-2018. *JAMA Health Forum.* 2022;3(8):e222663. doi:10.1001/jamahealthforum.2022.2663

eTable 1. Trends in naloxone out-of-pocket costs by payer

eTable 2. Trends in out-of-pocket costs by drug brand between 2010 and 2018 among the insured population

eTable 3. Trends in out-of-pocket costs by drug brand between 2010 and 2018 among the uninsured population

This supplemental material has been provided by the authors to give readers additional information about their work.

© 2022 Peet ED et al. *JAMA Health Forum.*

eTable 1. Trends in naloxone out-of-pocket costs by payer (table version of Figure 2)

		Claims (N)	Mean ($)	Lower CI ($)	Upper CI ($)
2010	Private	5,613	14.95	13.28	16.62
2011	Private	6,129	23.06	20.99	25.14
2012	Private	2,628	11.98	10.33	13.63
2013	Private	5,407	12.32	10.66	13.98
2014	Private	5,637	27.97	23.96	31.98
2015	Private	9,044	23.43	20.90	25.97
2016	Private	23,619	25.28	21.20	29.36
2017	Private	59,602	22.53	20.74	24.32
2018	Private	119,886	35.13	34.29	35.96
2010	Medicare	2,216	0.79	0.59	0.99
2011	Medicare	3,269	0.82	0.62	1.02
2012	Medicare	2,282	1.11	0.94	1.29
2013	Medicare	2,147	11.02	9.04	13.00
2014	Medicare	2,280	8.11	6.03	10.19
2015	Medicare	5,473	10.84	9.01	12.66
2016	Medicare	17,435	10.81	9.24	12.38
2017	Medicare	46,292	9.47	9.07	9.88
2018	Medicare	132,370	14.11	13.82	14.41
2010	Medicaid	8	0.16	-1.75	2.07
2011	Medicaid	4	3.33	3.33	3.33
2012	Medicaid	4	8.75	-2.61	20.10
2013	Medicaid	100	1.05	0.34	1.76
2014	Medicaid	783	3.14	1.97	4.30
2015	Medicaid	3,626	3.08	2.31	3.85
2016	Medicaid	18,680	3.23	2.90	3.56
2017	Medicaid	52,483	3.86	3.63	4.09
2018	Medicaid	118,881	2.85	2.70	3.00
2010	VA/Tricare				
2011	VA/Tricare	6	22.98	17.31	28.65
2012	VA/Tricare	143	17.49	16.68	18.30
2013	VA/Tricare	272	32.89	30.64	35.14
2014	VA/Tricare	750	103.07	88.35	117.78
2015	VA/Tricare	2,107	96.04	74.99	117.10

© 2022 Peet ED et al. *JAMA Health Forum.*

2016	VA/Tricare	6,035	59.34	43.86	74.82
2017	VA/Tricare	3,469	64.63	57.36	71.90
2018	VA/Tricare	6,303	72.67	67.35	77.98
2010	Uninsured	3,595	39.58	33.57	45.59
2011	Uninsured	6,072	76.01	70.15	81.87
2012	Uninsured	3,461	21.31	19.11	23.52
2013	Uninsured	5,322	23.59	21.09	26.09
2014	Uninsured	7,183	35.39	17.60	53.17
2015	Uninsured	6,226	94.23	78.06	110.39
2016	Uninsured	6,948	355.08	317.05	393.11
2017	Uninsured	7,013	301.03	273.00	329.05
2018	Uninsured	8,809	249.97	215.32	284.61

Note: Mean out-of-pocket costs in nominal dollars are defined by the total number of out-of-pocket costs per year divided by the total number of claims. Lower and upper confidence interval bounds presented reflect 95% confidence intervals. Additional details are provided in the "Methods" section

© 2022 Peet ED et al. *JAMA Health Forum.*

eTable 2. Trends in out-of-pocket costs by drug brand between 2010 and 2018 among the insured population (table version of Figure 4.A)

		Insured			
		Claims (N)	Mean ($)	Lower CI ($)	Upper CI ($)
2010	Naloxone	7,836	14.51	13.57	15.45
2011	Naloxone	9,885	14.98	13.85	16.11
2012	Naloxone	5,757	7.07	6.25	7.90
2013	Naloxone	7,927	7.72	6.61	8.83
2014	Naloxone	8,560	7.36	5.67	9.05
2015	Naloxone	13,584	7.22	4.05	10.38
2016	Naloxone	25,784	8.20	7.50	8.90
2017	Naloxone	26,119	7.20	6.69	7.72
2018	Naloxone	27,024	9.36	8.56	10.15
2010	Evzio				
2011	Evzio				
2012	Evzio				
2013	Evzio				
2014	Evzio	891	35.62	23.48	47.75
2015	Evzio	6,666	23.58	20.38	26.78
2016	Evzio	15,782	31.03	21.15	40.91
2017	Evzio	11,730	18.29	1.59	34.99
2018	Evzio	8,417	27.23	6.34	48.11
2010	Narcan				
2011	Narcan				
2012	Narcan				
2013	Narcan				
2014	Narcan				
2015	Narcan				
2016	Narcan	24,203	14.18	13.18	15.18
2017	Narcan	123,997	14.15	13.66	14.63
2018	Narcan	341,999	13.19	12.77	13.61

Note: Evzio is missing claims, means, and confidence intervals for the period 2010-2013 prior to its introduction. Similarly, Narcan is missing claims, means, and confidence intervals for the period 2010-2015 prior to its introduction. Mean out-of-pocket costs in nominal dollars are defined by the total number of out-of-pocket costs per year divided by the total number of claims. Lower and upper confidence interval bounds presented reflect 95% confidence intervals. Additional details are provided in the "Methods" section

© 2022 Peet ED et al. *JAMA Health Forum.*

eTable 3. Trends in out-of-pocket costs by drug brand between 2010 and 2018 among the uninsured population (table version of Figure 4.B)

		Uninsured			
		Claims (N)	Mean ($)	Lower CI ($)	Upper CI ($)
2010	Naloxone	3,596	46.07	40.04	52.11
2011	Naloxone	5,595	77.80	71.93	83.67
2012	Naloxone	2,761	21.91	19.70	24.12
2013	Naloxone	5,321	14.27	11.76	16.77
2014	Naloxone	6,922	73.89	65.73	82.04
2015	Naloxone	5,889	122.23	113.08	131.39
2016	Naloxone	5,099	72.88	68.58	77.18
2017	Naloxone	2,864	59.07	49.59	68.54
2018	Naloxone	2,371	18.44	15.90	20.98
2010	Evzio				
2011	Evzio				
2012	Evzio				
2013	Evzio				
2014	Evzio	260	51.75	-101.38	204.87
2015	Evzio	337	84.47	10.71	158.23
2016	Evzio	488	2136.37	1920.32	2352.42
2017	Evzio	364	1059.45	839.65	1279.26
2018	Evzio	215	1591.98	330.34	2853.62
2010	Narcan				
2011	Narcan				
2012	Narcan				
2013	Narcan				
2014	Narcan				
2015	Narcan				
2016	Narcan	1,361	87.95	80.27	95.63
2017	Narcan	3,785	89.70	85.45	93.95
2018	Narcan	6,223	60.70	56.46	64.95

Note: Evzio is missing claims, means, and confidence intervals for the period 2010-2013 prior to its introduction. Similarly, Narcan is missing claims, means, and confidence intervals for the period 2010-2015 prior to its introduction. Mean out-of-pocket costs in nominal dollars are defined by the total number of out-of-pocket costs per year divided by the total number of claims. Lower and upper confidence interval bounds presented reflect 95% confidence intervals. Additional details are provided in the "Methods" section

© 2022 Peet ED et al. *JAMA Health Forum.*
